# NBE-VLT-PFO: hybrid deep learning transformer architecture for joint estimation of lithium-ion batteries

**DOI:** 10.3389/frai.2026.1924449

**Published:** 2026-09-10

**Authors:** G. Tamizharasi, G. K. Rajini

**Affiliations:** School of Electrical Engineering, Vellore Institute of Technology University, Vellore, Tamil Nadu, India

**Keywords:** lithium-ion batteries, NBE-VLT, Polar Fox Optimization, SOC, SOH, and RUL

## Abstract

Lithium-ion batteries are utilized in electric vehicles (EVs), and accurate predictions of battery state of charge (SOC), state of health (SOH), and remaining useful life (RUL) are needed to ensure reliability, security, and durability of these batteries. This study provides a hybrid deep learning (DL) system, termed as Neural Basis Expansion Variational Ladder Transformer (NBE-VLT), integrating Polar Fox Optimization (PFO) for effective global hyperparameter tuning to produce accurate SOC, SOH, and RUL predictions from raw battery cycle data. The preprocessed battery cycle data were sent through a series of steps before the analysis to increase data features and reliability; these steps involved removing outliers, encoding features, normalizing the data, and aligning time. The Neural Basis Expansion (NBE) layer decomposes the battery's temporal signals using an adaptive basis function. The Variational Ladder Autoencoder (VLAE) generates hierarchical latent representations capable of learning and capturing degradation patterns and uncertainty information from battery data. In addition, a transformer encoder is used to learn long-range temporal dependencies, and an attention fusion mechanism combines features at different scales to produce accurate estimates of SOC, SOH, and RUL. The efficiency of the proposed model is shown through experimental evaluation conducted with the National Aeronautics and Space Administration (NASA) battery datasets, achieving SOC predictions with *R*^2^ of 0.9943, root mean squared error (RMSE) of 0.0225, mean absolute error (MAE) of 0.0164, and mean absolute percentage error (MAPE) of 1.64%. The SOH prediction achieved an R^2^ of 0.9606, RMSE of 0.0414, MAE of 0.0243, and MAPE of 2.43%, whereas RUL predictions achieved an *R*^2^ of 0.9936, RMSE of 0.0196, MAE of 0.0156, and MAPE of 1.56%. This indicates that the proposed framework, NBE-VLT-PFO, offers greater accuracy, robustness, and reliability for battery health forecasting.

## 1 Introduction

Due to the rapid growth of electric machines and the increased use of renewable energy sources by consumers, there is growing demand for better methods of storing energy from renewable sources. Lithium-ion battery technology is at the center of this transformation in energy storage for multiple applications (Qian et al., 2024). Electric vehicle (EV) and stationary energy storage applications show a strong trend toward lithium-ion battery technologies due to their numerous advantages. However, lithium-ion battery systems present significant challenges (Wang et al., 2024). As batteries are dynamic devices that experience variable loads, temperature, and charging conditions from external sources, they experience an accelerated rate of degradation and altered performance. Some measures on the batteries produce increased rates of degradation and create potentially hazardous conditions through thermal runaway (Liu et al., 2024; Gomathi, 2025).

Battery management systems (BMS) are critical to ensuring proper and safe operation of all lithium-ion battery systems due to the volatile nature of their use. Modern BMS designs require sophisticated algorithms capable of estimating the internal condition of the battery, which are capable of predicting the internal battery behavior under the diverse operating conditions encountered by the battery (Yao et al., 2024; Sakthimurugan et al., 2025).

Internal battery states such as state of charge (SOC), state of health (SOH), and remaining useful life (RUL) are crucial to battery management and computed from measurable quantities. Lithium-ion batteries are non-linear processes, creating complex relationships between observable quantities and battery state variables (Wei et al., 2025; Zhang et al., 2023; Wu et al., 2024). The time-varying nature of these systems, combined with the impact of operating circumstances, makes state estimation an inverse problem. More sources of uncertainty result from mature and cell-to-cell variations in battery pack characteristics (Babu et al., 2024; Fu et al., 2024).

Although electrochemical methods yield accurate physical and dynamic descriptions of battery systems, they are computationally expensive and not implementable in real time. Conversely, Equivalent Circuit Models (ECMs), such as Thevenin's or multi-resistor-capacitor (RC) type circuits, represent batteries using lumped electrical resistance, capacitance, and inductance with low complexity but limited accuracy compared to physical models. These types of models frequently use state observers, including extended Kalman filter (EKF), and unscented Kalman filter (UKF) (Lin et al., 2025; Zhang et al., 2025; Chen et al., 2024). However, because of the inherent complexities within model-based techniques, they experience significant parameter sensitivity, little robustness to aging, and limited ability to identify strong non-linearity and heterogeneous operation (Xie et al., 2023).

To address the problems of model-based methods, data-driven methods using ML and artificial intelligence (AI) have been thoroughly researched. Data-driven methods are designed to fit a non-linear mapping from measurable quantities to the battery's internal state without an explicit physical model (Shen et al., 2024; Fang et al., 2023). There are many different classification algorithms that are successfully used in estimating SOC and RUL. Recently deep learning (DL) architectures have been found to be useful in identifying temporal dependencies and non-linear degradation patterns. However, data-driven methods present their own challenges, including issues with dataset heterogeneity, interpretation of features, ability to generalize to new data, and potential for information leakage due to the correlation between the target and derived features in a controlled experimental environment (Mu et al., 2025; Zhao et al., 2023). Table 1 represents the summary of related models.

**Table 1:** Related works of existing models for SOC, SOH, and RUL predictions.

S. No.	References	Methods	Merits	Demerits	Metrics
1	Gao et al. (2023)	Convolutional Neural Networks—Bidirectional Long Short-Term Memory (CNN-BiLSTM)	CNN-BiLSTM model can extract local features of batteries effectively through the use of CNN and identify long-term temporal dependencies through the use of BiLSTM networks.	It has a high degree of computational complexity and takes a long time to train.	MAE: 0.5930, RMSE: 0.8306, *R*^2^: 0.957
2	Krishnasamy and Muniandi (2026)	Random Forest—Long Short-Term Memory—Extreme Gradient Boosting (RF-LSTM-XGBoost)	The integration enables improved feature learning, temporal modeling, and more robust prediction.	The combination of models results in increased implementation complexities and requires numerous hyperparameter tuning processes to complete.	MAE: 0.025, RMSE: 0.035, *R*^2^: 0.96
3	Li and Chen (2025)	Long Short-Term Memory—Particle Swarm Optimization (LSTM-PSO)	PSO automatically tunes the hyperparameters of LSTM models, thereby improving prediction accuracy and reducing effort.	It is computationally expensive to run the optimization process, which requires multiple iterations before optimal performance is achieved.	MAE: 0.67, RMSE: 0.94, *R*^2^: 0.929
4	Li et al. (2025)	Sparrow Search Algorithm—Long Short-Term Memory (SSA-LSTM)	The SSA optimizes the hyperparameters for LSTM, leading to improved convergence behavior and more stable predictions of battery states.	Execution times and resource demands are larger than the single methods.	MAE: 0.517, RMSE: 0.708, *R*^2^: 0.956
5	Dini and Paolini (2026)	Extra trees	Extra Trees offers rapid training, good generalization ability, and high resistance to noise in battery-generated data due to its ensemble learning strategy.	Its lack of explicit modeling of temporal dependencies leads to a reduced ability to capture the trend in degradation over time.	MAE: 0.0173, RMSE: 0.0367, *R*^2^: 0.982
6	Chen et al. (2023)	Genetic Algorithm—Support Vector Regression (GASVR)	It demonstrates improved non-linear regression performance and superior estimation accuracy when training data is limited.	Due to a large-sized time-series battery dataset, it is expensive in computational tasks.	MAE: 0.77, RMSE: 0.95, *R*^2^: 0.9892

To tackle these problems, a proposed Neural Basis Expansion Variational Ladder Transformer integrating Polar Fox Optimization (NBE-VLT-PFO) framework is used for hierarchical latent feature learning to give more robust results through improved generalization of transformer-based temporal modeling and to reduce the risk of overfitting by performing hyperparameter optimization using PFO.

### 1.1 Major contributions

The primary contributions of this research include the following:

- In preprocessing, to increase data quality, reduce noise, and improve battery degradation model stability, some methods are applied, such as encoding features, eliminating outliers, including relevant features, and normalizing features.
- The NBE-VLT model is designed to perform well in learning non-linear dependencies of lithium-ion batteries, producing precise estimation.
- PFO is allowed for faster convergence and less manual hyperparameter tuning by improving accuracy and generalization for forecasting.

## 2 Proposed system description

**Figure 1:**
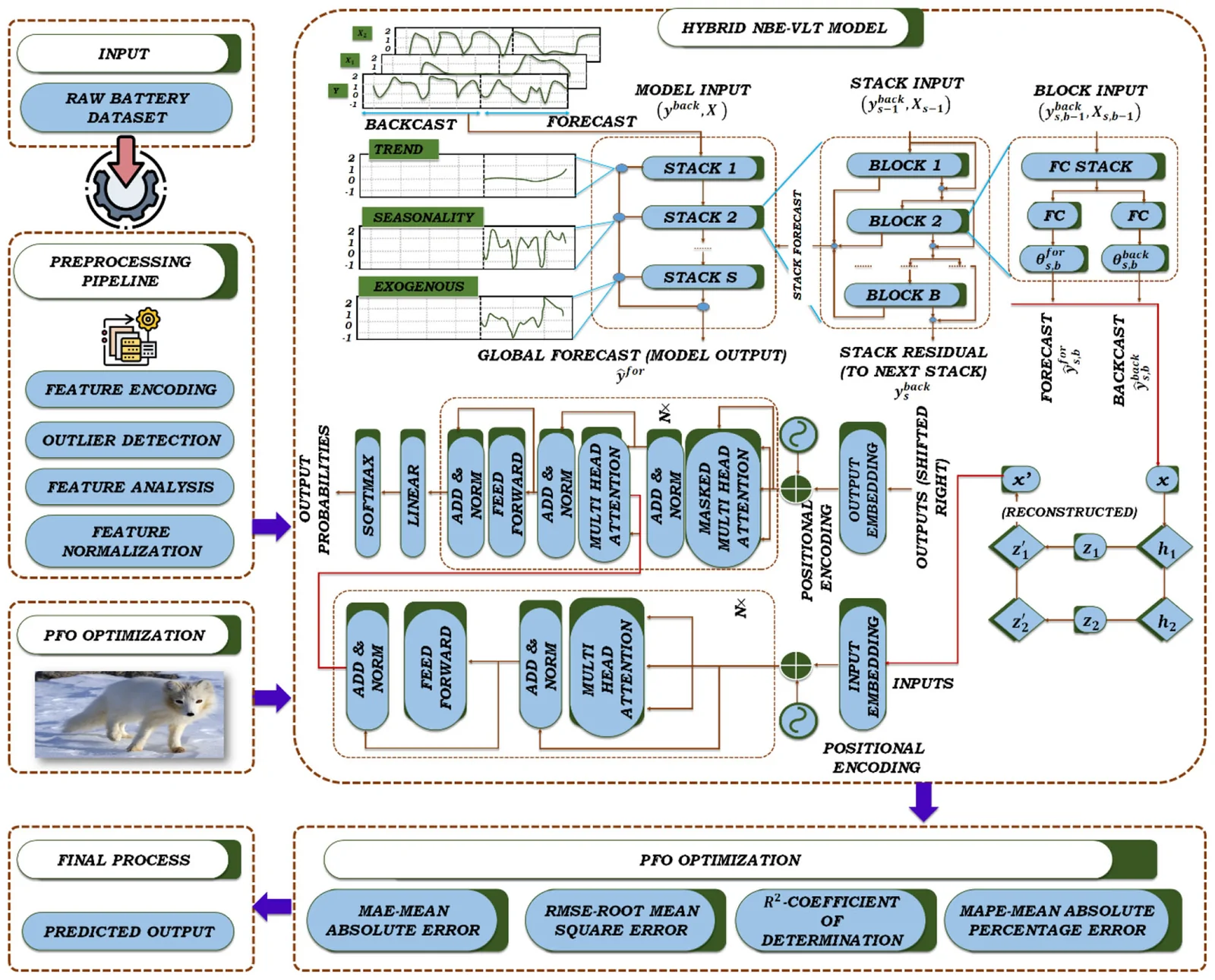
Overall proposed framework.

The Hybrid NBE-VLT-PFO framework is a complete DL architecture that is developed for estimating lithium-ion battery parameters. The structure of the suggested system is displayed in Figure 1.

These parameters include SOC, SOH, and RUL. The process starts with a raw battery dataset, followed by a series of preprocessing steps, including feature encoding, outlier detection, assessing feature relevance to the task using statistical methods, and applying normalization to ensure the data are clean, relevant, and standardized before being fed into the model. The base model uses techniques such as NBE to learn non-linear degradation patterns, Variational Ladder Encoding to create a hierarchical latent representation of an observation and capture uncertainty in the built-in model, and a Transformer Encoder to learn the temporal dependencies between the batteries throughout their mission life. An attention fusion method is used to combine information from all features to create robust latent embeddings for all observed battery cycles. PFO automatically tunes hyperparameters, such as learning rate, latent dimensions, and network depth, to improve convergence, stability, and predictive capabilities, enabling accurate and efficient modeling of complex battery behavior across a wide range of operational conditions.

## 3 Proposed system modeling

### 3.1 Preprocessing

#### 3.1.1 Feature encoding

**Figure 2:**
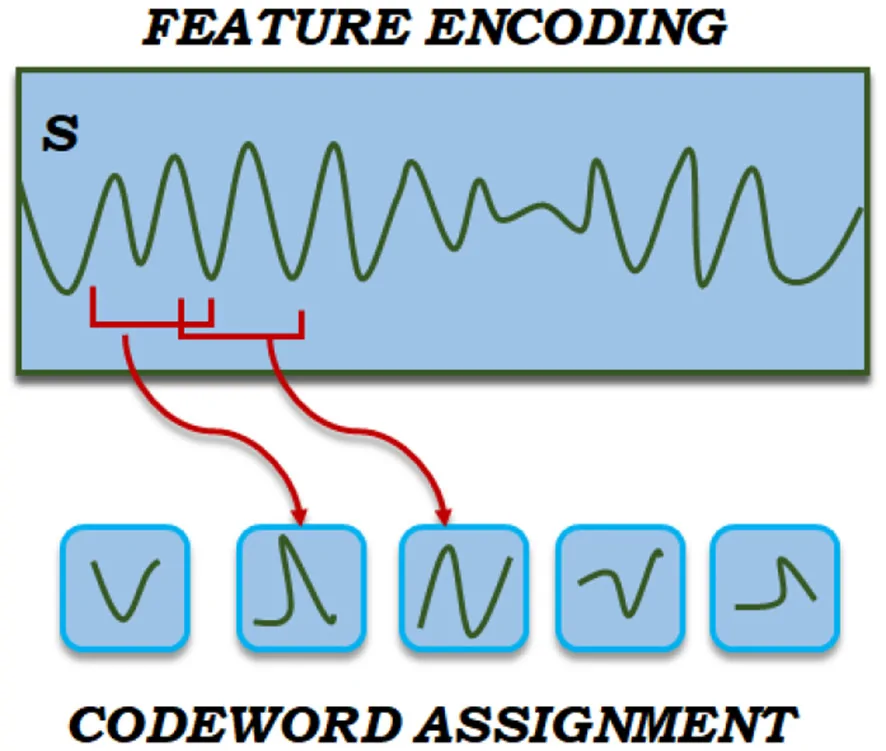
Feature encoding.

To predict lithium-ion battery condition, large amounts of charge and discharge data are compressed into a small set of representative features using a feature encoding technique. The diagram is presented in Figure 2.

The encoding coefficients are calculated to minimize reconstruction error while preserving local relationships among battery features. Equation (1) represents the encoding coefficient vector


$$\begin{array}{l} v=\arg\underset{v}{\min}||x-Cv{||}_{2}^{2}+\lambda ||d⊙v{||}_{2}^{2},\;{s.t.1}^{M}v=1 \end{array}$$ (1)

Here, *x* is the feature vector for the extracted battery feature, *C* is the codebook of features, *v* is the encoding coefficient vector, λ is a regularization coefficient, and *d* is the locality adopter. This process reduces feature redundancy while obtaining representative encoded features that capture critical battery degradation and performance characteristics.

#### 3.1.2 Outlier detection

Detection of outliers is a crucial component in estimating lithium-ion batteries. Outlier detection identifies data points that are significantly different from the majority of the data. The outlier data points stem from operational differences across testing conditions, age-related effects on lithium-ion batteries, or an atypical lithium-ion battery type. Developing a clearer view of battery performance by identifying outlier data points assists in more accurate estimation of key considerations. Furthermore, the analysis of unusual observations produces useful information about their respective battery characteristics and degradation profile, which in turn ultimately contribute to building more robust battery management systems and monitoring systems. In determining the outliers, the Interquartile range (IQR) procedure is applied, whereby data points that fall outside the range are excluded.

#### 3.1.3 Feature analysis

Feature analysis examines the voltage, current, temperature, capacity, internal resistance, and cycle count associated with a set of batteries to assist in studying battery behavior based on these characteristics. It discovers the most pertinent information needed to estimate the battery's three primary states. Feature analysis improves the reliability and efficiency of lithium-ion battery estimation models. Pearson correlation analysis is used to assess feature importance, whereby only highly correlated features are used to train the model.

#### 3.1.4 Feature normalization

An important step in lithium-ion battery estimation is the normalization of features in the dataset. Several battery-related features have different ranges and units. The most effective method to normalize these features is to perform Minimum–Maximum (Min–Max) normalization, which is accomplished by scaling the original values of each feature in the dataset and transforming them into a new set of values that fall within the common range of [0,1]. Min–Max normalization equation is given in Equation (2):


$$\begin{array}{l} x_{transformed}=\frac{x-min\left(x\right)}{max\left(x\right)-min\left(x\right)} \end{array}$$ (2)

Normalizing all features to a common range allows Min–Max normalization to improve learning and create more accurate estimates of key characteristics.

### 3.2 Model training

#### 3.2.1 Neural basis expansion variational ladder transformer (NBE-VLT)

The proposed NBE-VLT structure comprises two main components: NBE modules and VLT, which together enable non-linear understanding of the relationship between empirical data and estimated states. Initially, NBE learns to extract the temporal and spatial correlations in the time-series signals of a lithium-ion battery to construct the battery cells' time-series using a combined approach that decomposes the time-series database into basis functions. Let *Y*^*back*^ be the historical measurements of batteries. For block *b* of stack *s*, a fully connected neural network learns to extract and represent hidden features of block *s*. The relevant formulations are provided in Equations (3–16)


$$\begin{array}{l} h_{s,b}=FCNN_{s,b}\left(Y_{s,b-1}^{back},X_{b-1}\right) \end{array}$$ (3)

Let $Y_{s,b-1}^{back}$ denote the residual backcast of battery cells from the previous block, *X*_*b*−1_ represent the exogenous battery variables for block *s* of time *t*+1, and *h*_*s, b*_ is defined as learned hidden representations. Finally, learned hidden representations of neural networks in block *b* from all stacks are used to create the expansion coefficients for battery cells.


$$\begin{array}{l} \theta_{s,b}^{back}=LINEAR^{back}\left(h_{s,b}\right) \end{array}$$ (4)

$$\begin{array}{l} \theta_{s,b}^{for}=LINEAR^{for}\left(h_{s,b}\right) \end{array}$$ (5)

**Figure 3:**
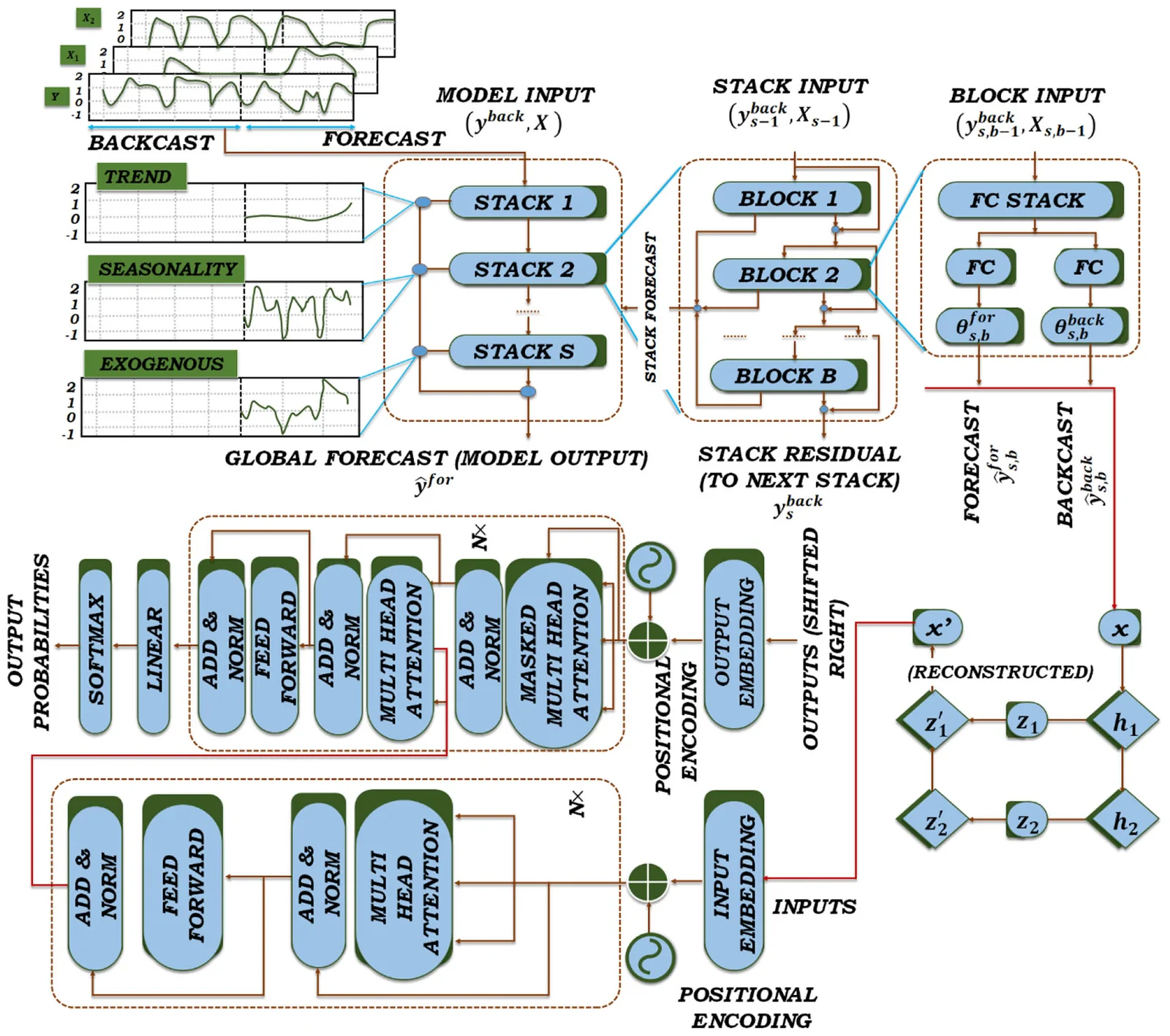
Model of NBE-VLT.

Here, $\theta_{s,b}^{back}$ represents a backcast expansion coefficient and $\theta_{s,b}^{for}$ represents a forecast expansion coefficient. The learned coefficients are expanded into their respective basis vectors to reconstruct the backcast and create the forecast representation.


$$\begin{array}{l} {Ŷ}_{s,b}^{back}=V_{s,b}^{back}\theta_{s,b}^{back} \end{array}$$ (6)

$$\begin{array}{l} {Ŷ}_{s,b}^{for}=V_{s,b}^{for}\theta_{s,b}^{for} \end{array}$$ (7)

where $V_{s,b}^{back}$ and $V_{s,b}^{for}$ denote the backcast and forecast basis matrices. The process of basis expansion decomposes battery performance into temporal components that are described independently of one another according to degradation and operational dynamics. The framework of NBE-VLT is displayed in Figure 3.

The use of residual stacking helps to eliminate the explained components of the signal and improves the extraction of features, namely:


$$\begin{array}{l} Y_{s,b+1}^{back}=Y_{s,b}^{back}-{Ŷ}_{s,b}^{back} \end{array}$$ (8)

$$\begin{array}{l} {Ŷ}_{s}^{for}=\overset{B}{\underset{b=1}{\sum }}{Ŷ}_{s,b}^{for} \end{array}$$ (9)

where, *B* is the block count within a stack. The final NBE representation is generated by aggregating all outputs from the different stacks. This number of stacks provides a compact and interpretable representation of battery degradation over time.


$$\begin{array}{l} {\hat{Y}}^{for}=\overset{S}{\underset{S=1}{\sum }}{Ŷ}_{s}^{for} \end{array}$$ (10)

The total number of battery stacks is denoted by *S*, the overall stack number, to provide a compact and interpretable framework for representing battery degradation trajectories through additive decomposition. To encode these basis-expansion-extracted features, VLAE is employed. Regularization of the latent distribution is performed to prevent the collapse of latent variables and to derive interpretable hierarchical representations using the Maximum Mean Discrepancy (MMD) criterion.


$$\begin{array}{r} MMD\left(q\left(z\right),p\left(z\right)\right)=Eq\left(z\right),q\left(z_{0}\right)\left[k\left(z,z_{0}\right)\right]\\ +Ep\left(z\right),p\left(z_{0}\right)\left[k\left(z,z_{0}\right)\right]-2Ep\left(z\right),q\left(z_{0}\right)\left[k\left(z,z_{0}\right)\right] \end{array}$$ (11)

An expectation operator (*E*[·]) and kernel function (*k*(·)) are included in the definition of the MMD distance of the posterior distribution (*q*(*z*)) relative to the prior distribution (*p*(*z*)) is defined as:


$$\begin{array}{l} k\left(z,z^{\prime }\right)=e^{-||z-z^{\prime }{||}_{2}^{2}/\sigma^{2}} \end{array}$$ (12)

The MMD distance is calculated using a Gaussian kernel function based on the original formulation and σ is the kernel bandwidth.


$$\begin{array}{r} LMMD-VLAE=Eq_{\varphi }\left(z|x\right)\left[\log_{p_{\theta }}\left(x|z\right)\right]\\ -MMD\left(P\left(z_{0}\right),q_{\varphi }\left(z_{0}\right)\right)-MMD\left(P\left(z_{1}\right),q_{\varphi }\left(z_{1}\right)\right) \end{array}$$ (13)

MMD is preferred over KL divergence since it offers better flexibility in distribution matching and a lower chance of posterior collapse. Thus, VLAE is capable of learning expressive latent representations enabling to extract the information of non-linear degradation characteristics of lithium-ion batteries more efficiently. Therefore, the resulting MMD-VLAE is defined by the two respective distributions, the encoder posterior distribution *q*_φ_(*z*_1_) and decoder function *p*_θ_(*z*_1_), in addition to both encoder and decoder parameters, the hierarchical latent representation exhibits both short-term operational characteristics and long-term degradation mechanisms.

To characterize long-term temporal dependencies in the data, the hierarchical latent structures are processed by a Transformer module. The input to the Transformer is the scaled dot-product attention, which is calculated as follows:


$$\begin{array}{l} Attention\left(Q,K,V\right)=softmax\left(\frac{QK^{T}}{\sqrt{d_{k}}}\right)V \end{array}$$ (14)

where *d*_*k*_ is the dimension of the key vector. This model dynamically focuses on the most relevant temporal states contributing to battery degradation. Multi-head attention is used to capture different degradation patterns.


$$\begin{array}{l} MultiHead\left(Q,K,V\right)=Concat\left(head_{1},.\;.\;.,head_{h}\right)W^{O} \end{array}$$ (15)

where,


$$\begin{array}{l} head_{i}=Attention\left(QW_{i}^{Q},KW_{i}^{K},VW_{i}^{V}\right) \end{array}$$ (16)

Here *h* indicates the number of heads, $W_{i}^{Q}$, $W_{i}^{K},$ and $W_{i}^{V}$ are the learned projections for queries, keys, and values, respectively, and *W*^*O*^ is the output projection. Thus, the method learns about diverse behaviors of the battery such as thermal behavior, voltage recovery, charge transfer, and long-term capacity degradation.

By combining NBE, VLAE, and a Transformer attention model, where NBE obtains non-linear degradation features and multi-scale battery patterns, VLAE acquires hierarchical latent representations and uncertainty information, and Transformer captures long-term temporal dependencies via self-attention. The modules are integrated sequentially to efficiently perform feature extraction, representation learning, and temporal modeling, thereby improving the accuracy of SOC, SOH, and RUL estimation.

### 3.3 Hyperparameter tuning

#### 3.3.1 Polar Fox Optimization (PFO)

PFO employs a bio-inspired heuristic optimization method to optimize estimation, inspired by the adaptive hunting, survival, and group behaviors that Arctic foxes display when hunting together. As such, their adaptive behaviors allow the algorithm to maintain a balance between exploration and exploitation while conducting an optimization search. The optimization starts by randomly defining a population of potential candidate solutions. Equations (17–26) represent the relevant expressions of PFO

Each candidate solution, *x*_*ij*_, is created within the search limits by the following equation:


$$\begin{array}{l} x_{ij}=LB+r_{1}.\left(UB-LB\right) \end{array}$$ (17)

where *LB* and *UB* are the search space's lower and upper bounds, and *r*_1_ is a randomly generated value from the range of 0 to 1. After this initial population is created, the potential candidates are divided into different behavioral groups, and each behavioral group's weights continue to be modified through an iterative updating process. The group weighting update rule is given by the following equation:


$$\begin{array}{l} W_{i}^{new}=W_{i}+\frac{t^{2}}{NG_{i}} \end{array}$$ (18)

where *t* indicates the present repetition and *NG*_*i*_ is the sum of members of the group *i*. Subsequently, each candidate solution updates its position based upon its previous experience. The following equations determine this new position and mimic the ability of the fox to find its prey based upon its previous hunting experiences.

The update of position, the calculation of the jump force (*P*) and the movement direction (*D*) is as follows:


$$\begin{array}{l} x_{i}^{t+1}=x_{i}^{t}+PD \end{array}$$ (19)

$$\begin{array}{l} P=r_{2}PF_{i} \end{array}$$ (20)

$$\begin{array}{l} D=\cos\left(r_{3}\right) \end{array}$$ (21)

**Figure 4:**
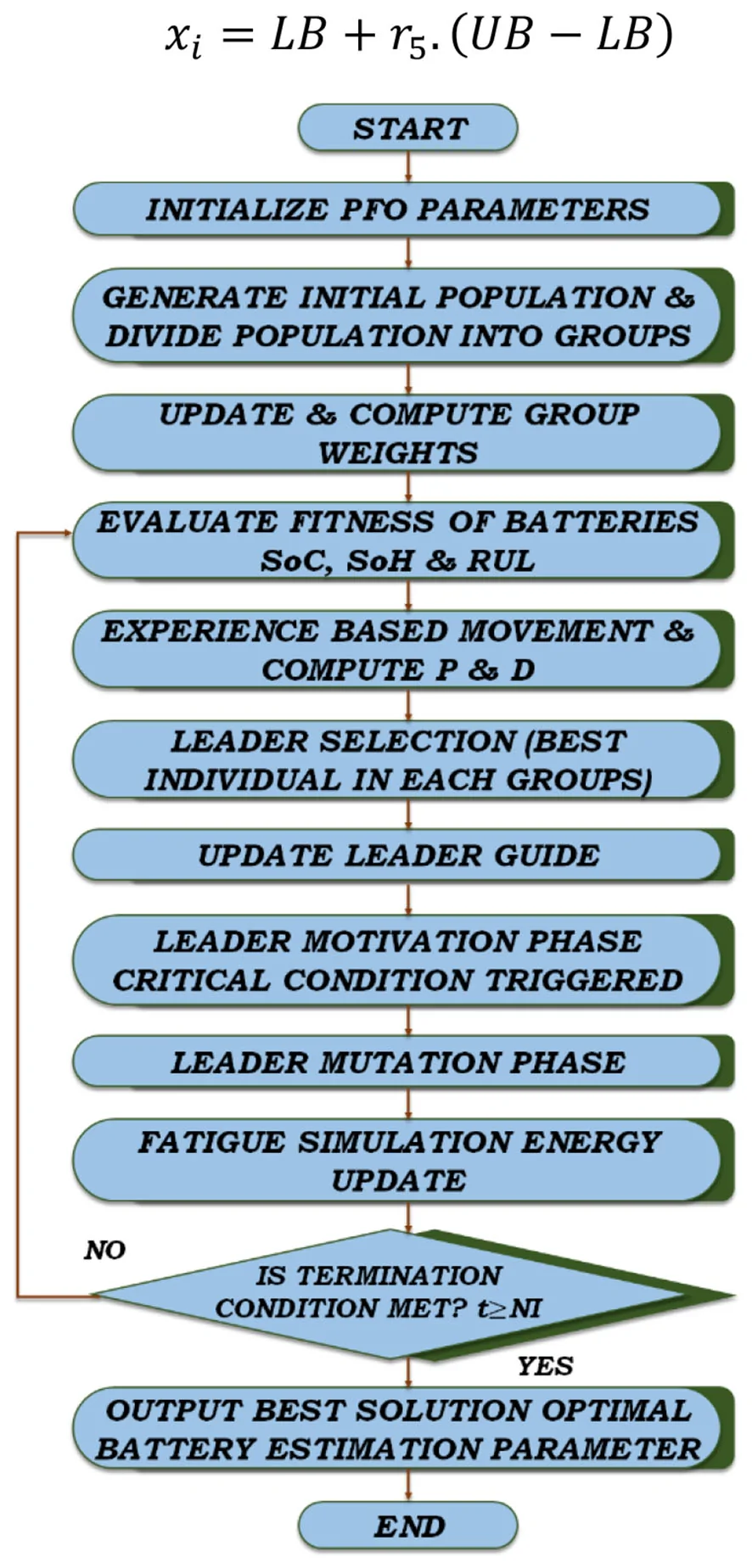
Flowchart of PFO.

In this equation, *PF*_*i*_ indicates the strength of the person based on their prior experience, while *r*_2_ and *r*_3_ are random numbers. Within the subgroup, the best-performing person is the group leader, while others update their positions based on the leader's location. This is presented as follows:


$$\begin{array}{l} x_{i}^{t+1}=x_{i}^{t}+r_{4}.\frac{\left(x_{i}^{t}-L\right)}{LF_{i}} \end{array}$$ (22)

where *L* is the leader location, *LF*_*i*_ is the leader influence factor, and *r*_4_ is a random number between −1 and 1. A motivational factor for the leader is undermined if the members detect no improvement at all. This condition is expressed as follows:


$$\begin{array}{l} critical=\left(NLM\left[MLM\right]\right)\vee \left(\frac{t}{NI}\geq 0.8\right) \end{array}$$ (23)

where *NLM* is the leader motivation number, *MLM* is the predetermined threshold, *t* is the current iteration, and *NI* is the extreme number of iterations permitted. Through mutation, the poorly performing individuals are replaced, adding diversity to the overall population. The flowchart of PFO is shown in Figure 4. New location coordinates of the individuals are computed as follows:


$$\begin{array}{l} x_{i}=LB+r_{5}.\left(UB-LB\right) \end{array}$$ (24)

where *r*_5_ is a random number between 0 and 1. The algorithm simulates the effect of exhaustion on group members by decreasing the group's total energy after each iteration. The energy use for group *G*_1_ is as follows:


$$\begin{array}{l} G_{1}=min\left(G_{1}-G_{1r},G_{1i}\right) \end{array}$$ (25)

If there are few members in the group, the energy is reset to ensure the following expression achieves stability, and it is expressed as follows:


$$\begin{array}{l} G_{1}=G_{m} \end{array}$$ (26)

Algorithm 1 represents the pseudo code of PFO. Table 2 denotes the optimized values of PFO. The PFO algorithm provides an overall solution for improving candidate solutions by continually updating, learning from others in the group, and adapting. Due to these aspects, the PFO algorithm finds optimal parameter values for lithium-ion battery estimation models, significantly increasing the accuracy of predicting the battery's SOC, SOH, and RUL.

Algorithm 1 Pseudocode: PFO.

  START
  Initialize population size N, MaxIter, bounds LB
and UB
  Initialize fox positions Xi randomly within bounds
  Evaluate fitness of all foxes
  Set best fox Xbest based on fitness
  FOR iter = 1 to MaxIter DO
      FOR each fox i = 1 to N DO
        Generate random parameters α, β, γ
        Compute exploration movement Ei
        Compute exploitation movement Si
        Update position:
        Xi_new = Xi + α^*^Ei + β^*^Si + γ^*^random_walk
        IF Xi_new < LB THEN
          Xi_new = LB
        ELSE IF Xi_new > UB THEN
          Xi_new = UB
        END IF
        Evaluate fitness of Xi_new
        IF fitness(Xi_new) < fitness(Xi) THEN
          Xi = Xi_new
        END IF
        IF fitness(Xi) < fitness(Xbest) THEN
          Xbest = Xi
        END IF
       END FOR
      RETURN Xbest
  END

**Table 2:** Optimized values of PFO.

S. no.	Parameter name	Value
1	Population size	30
2	Maximum iterations	5
3	Lower bound	−10
4	Upper bound	10
5	Exploration factor	1.0
6	Exploitation factor	1.0
7	Random walk coefficient	0.5
8	Adaptive control parameter	0.3
9	Inertia weight	0.7
10	Stopping criterion	1e−6

## 4 Results and discussion

The proposed model integrates an architecture based on transformers, DL, and PFO to effectively reflect both temporal relationships and non-linearity associated with battery behavior. Table 3 provides a description of the dataset used in the proposed system. Table 4 denotes the samples in NASA battery dataset grouped by battery ID.

**Table 3:** Dataset description.

Dataset name	NASA battery dataset
Number of samples	29,180
Number of features	8
Target features	SOC, SOH, and RUL

**Table 4:** Samples in NASA battery dataset grouped by battery ID.

S. no.	Battery ID	Samples
1	B0036	3,920
2	B0007	3,340
3	B0005	2,460
4	B0033	2,300
5	B0006	2,180
6	B0018	2,080
7	B0034	1,400
8	B0042	1,140
9	B0043	1,080
10	B0044	1,000
11	B0030	800
12	B0031	800
13	B0032	800
14	B0029	800
15	B0038	700
16	B0039	600
17	B0040	660
18	B0027	560
19	B0025	560
20	B0028	560
21	B0026	540
22	B0046	340
23	B0048	280
24	B0047	220

**Figure 5:**
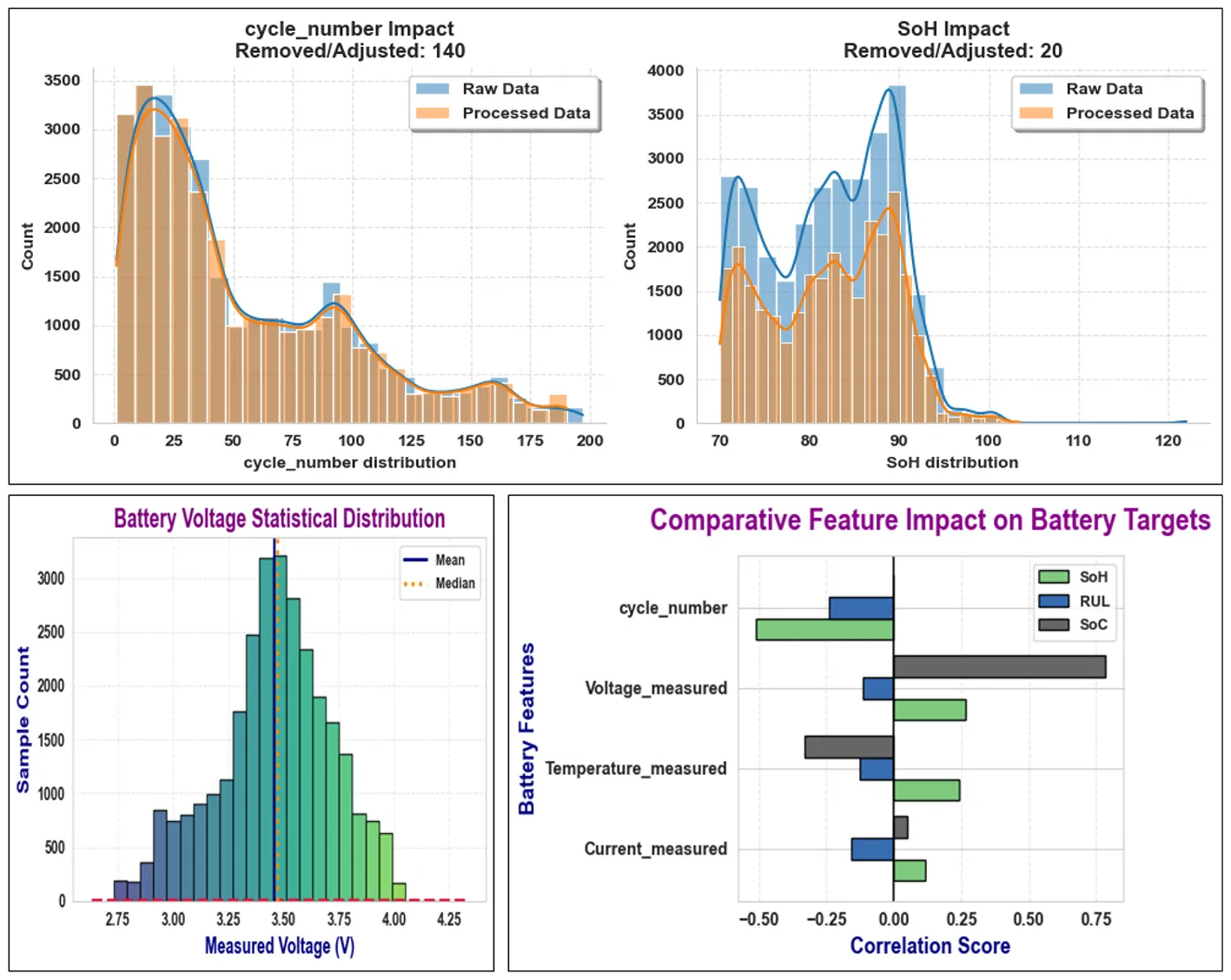
Battery data visualization.

Figure 5 illustrates the steps in preprocessing battery data and analyzing the selected features. It depicts the distributions of cycle numbers and SOH before and after data processing; here, outliers (140 cycle numbers and 20 SOH values) are removed to achieve a higher quality dataset. The voltage distribution approximates a normal distribution additionally with closely matching mean and median values. The feature impact analysis demonstrates that cycle number has the greatest effect on SOH, charge voltage has the strongest correlation with SOC, whereas temperature and current have a moderate effect across different states of charge for the six batteries tested.

The ablation study in Table 5 establishes that each added component improves SOC estimation performance. The proposed Hybrid NBE-VLT-PFO model achieves the maximum *R*^2^ (0.9943) and the minimum RMSE (0.0225), MAE (0.0164), and MAPE (1.64%), indicating high precision in estimating different variables.

**Table 5:** Ablation study for SOC estimation.

Model	*R* ^2^	RMSE	MAE	MAPE (%)
NBE	0.9648	0.0584	0.0432	4.31
NBE-VLAE	0.9759	0.0468	0.0346	3.47
NBE-transformer	0.9798	0.0417	0.0305	3.06
NBE-VLT	0.9850	0.0360	0.0260	2.63
Hybrid NBE-VLT-PFO (proposed)	0.9943	0.0225	0.0164	1.64

The ablation study in Table 6 shows that each component increases SOH estimation performance. The Hybrid NBE-VLT-PFO model achieves the maximum *R*^2^ (0.9606) and the minimum RMSE (0.0414), MAE (0.0243), and MAPE (2.43%) values, making it more accurate in in estimating the SOH value.

**Table 6:** Ablation study for SOH estimation.

Model	*R* ^2^	RMSE	MAE	MAPE (%)
NBE	0.8918	0.0728	0.0496	4.98
NBE-VLAE	0.9075	0.0649	0.0438	4.41
NBE-transformer	0.9152	0.0608	0.0405	4.06
NBE-VLT	0.9240	0.0570	0.0380	3.81
Hybrid NBE-VLT-PFO (proposed)	0.9606	0.0414	0.0243	2.43

**Figure 6:**
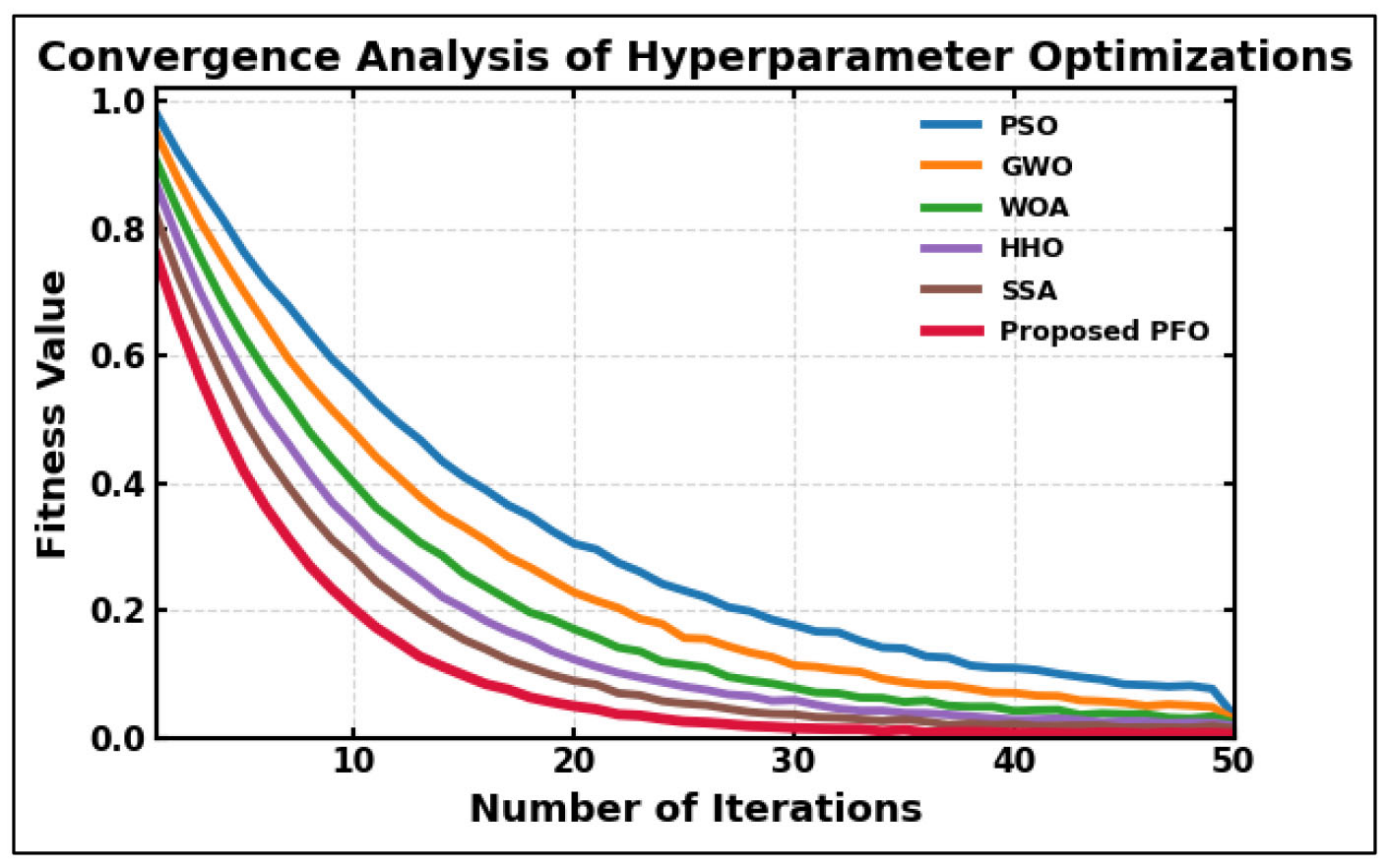
Convergence analysis of hyperparameter optimization.

The convergence analysis in Figure 6 indicates that the proposed PFO converges quickly and achieves a lower fitness value than other techniques such as PSO, GWO, WOA, HHO, and SSA, which shows effective optimization performance and fast hyperparameter tuning.

The ablation study in Table 7 shows that each component enhances RUL estimation performance. The Hybrid NBE-VLT-PFO model achieves the best performance in terms of R^2^ value (0.9936) and the lowest calculated values of RMSE (0.0196), MAE (0.0156), and MAPE (1.56%), indicating reliable operation.

**Table 7:** Ablation study for RUL estimation.

Model	*R* ^2^	RMSE	MAE	MAPE (%)
NBE	0.9524	0.0598	0.0413	4.12
NBE-VLAE	0.9646	0.0497	0.0335	3.35
NBE-transformer	0.9713	0.0438	0.0286	2.87
NBE-VLT	0.9780	0.0370	0.0240	2.37
Hybrid NBE-VLT-PFO (proposed)	0.9936	0.0196	0.0156	1.56

**Figure 7:**
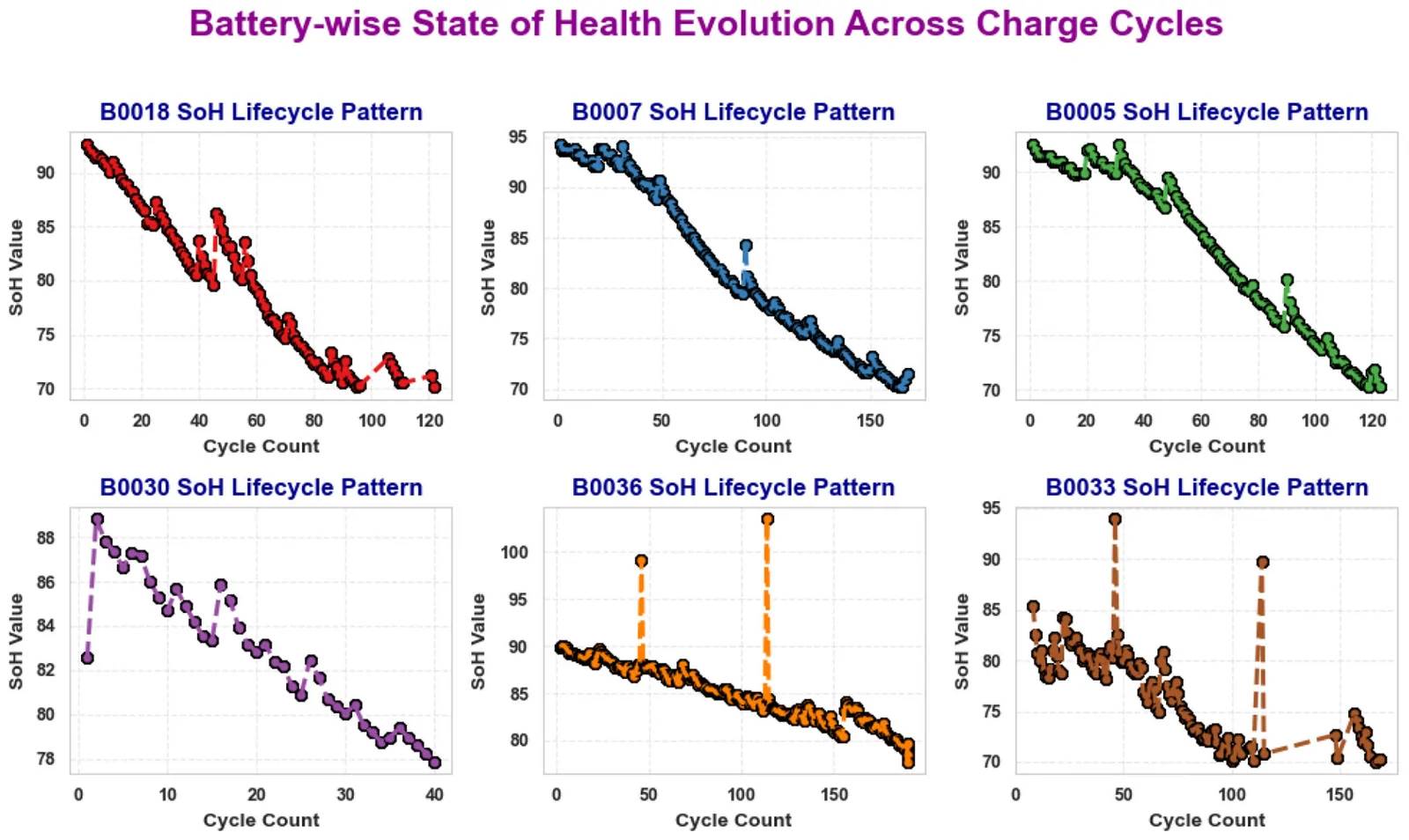
Battery-wise SOH evolution across charge cycles.

Figure 7 shows that the SOH of all six batteries decreases with increasing total charge cycles, indicating this is typical of battery degradation over time, although some batteries exhibit small fluctuations in their SOH values. In certain critical cycle moments, the SOH gains are caused by the effects of capacity recovery, measurement noise, or changes in conditions of operation. The proposed model (NBE-VLT-PFO) handles non-monotonic behavior by enabling recognition of various degradation processes and time dependencies, thereby calculating SOH reliably.

**Figure 8:**
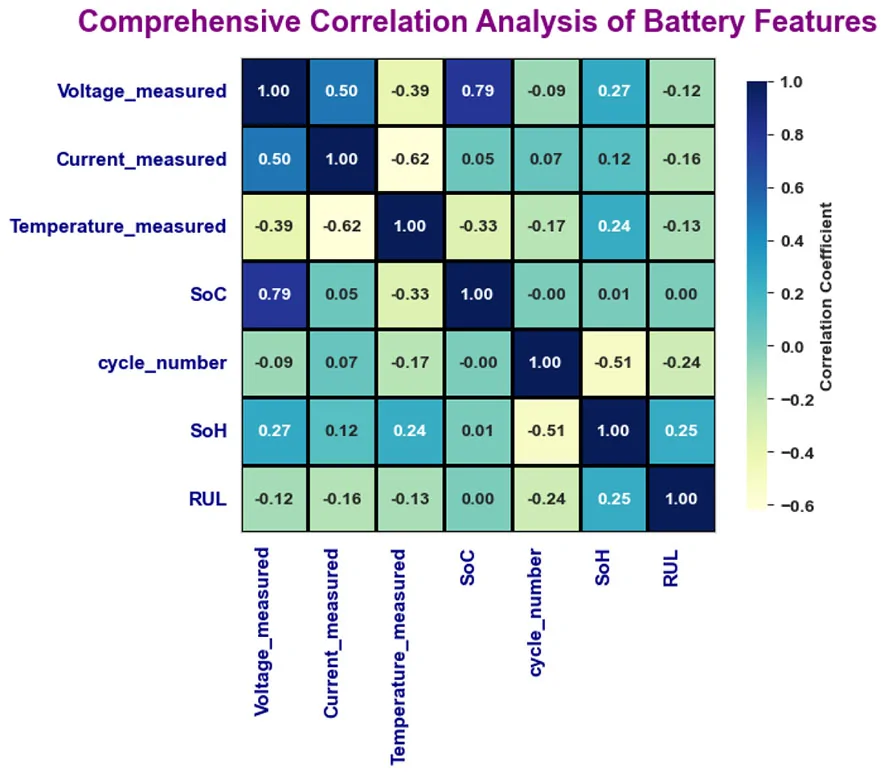
Comprehensive correlation analysis of battery features.

In Figure 8, the heat map illustrates the degree of correlation among variables, highlighting a strong positive correlation (0.79) between charge voltage and SOC (−0.51) between cycle number and SOH, and (0.25) between RUL and SOH. In summary, the heat map captures the primary interdependencies among variables related to electrical performance, temperature behavior, and aging.

In Table 8, the comparison of the optimizers reveals that the proposed PFO obtains the greatest outcomes with the minimum fitness value (0.0052) and minimum execution time (114 s), as well as having the highest prediction accuracy with the *R*^2^ values 0.9943, 0.9606, and 0.9936, respectively, compared to PSO, GWO, WOA, HHO, and SSA optimizers.

**Table 8:** Comparison of optimization.

Optimizer	Best fitness	SOC *R*^2^	SOH *R*^2^	RUL *R*^2^	Time (s)
PSO	0.0335	0.9881	0.9412	0.9827	148
GWO	0.0278	0.9894	0.9469	0.9853	136
WOA	0.0224	0.9906	0.9517	0.9875	129
HHO	0.0186	0.9920	0.9558	0.9902	123
SSA	0.0137	0.9932	0.9584	0.9921	118
Proposed PFO	0.0052	0.9943	0.9606	0.9936	114

**Figure 9:**
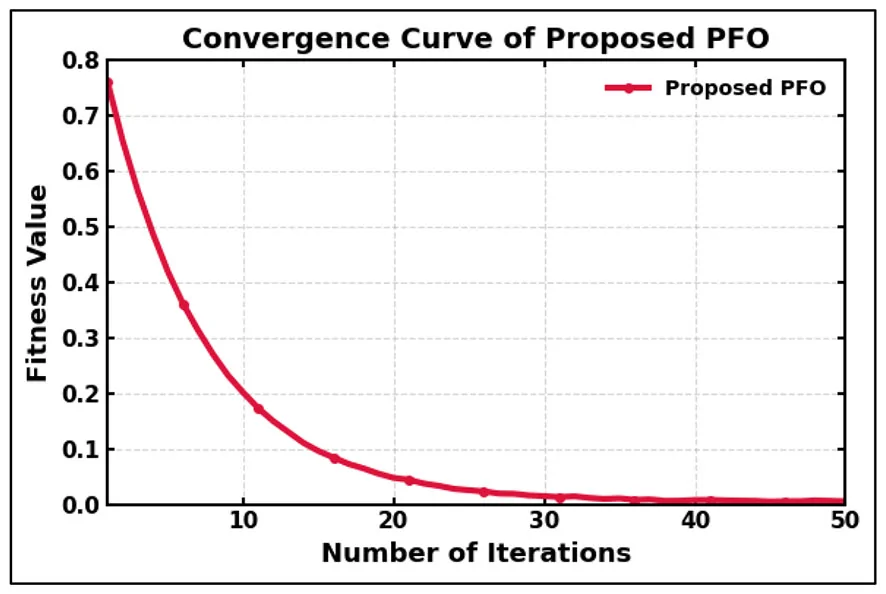
Convergence curve of the proposed PFO.

The convergence plot in Figure 9 of the proposed PFO shows a considerable reduction in fitness value in the first iterations, followed by a steady approach toward the optimal solution. The fitness value is close to zero, which indicates a high convergence rate and efficiency of the suggested PFO algorithm.

**Figure 10:**
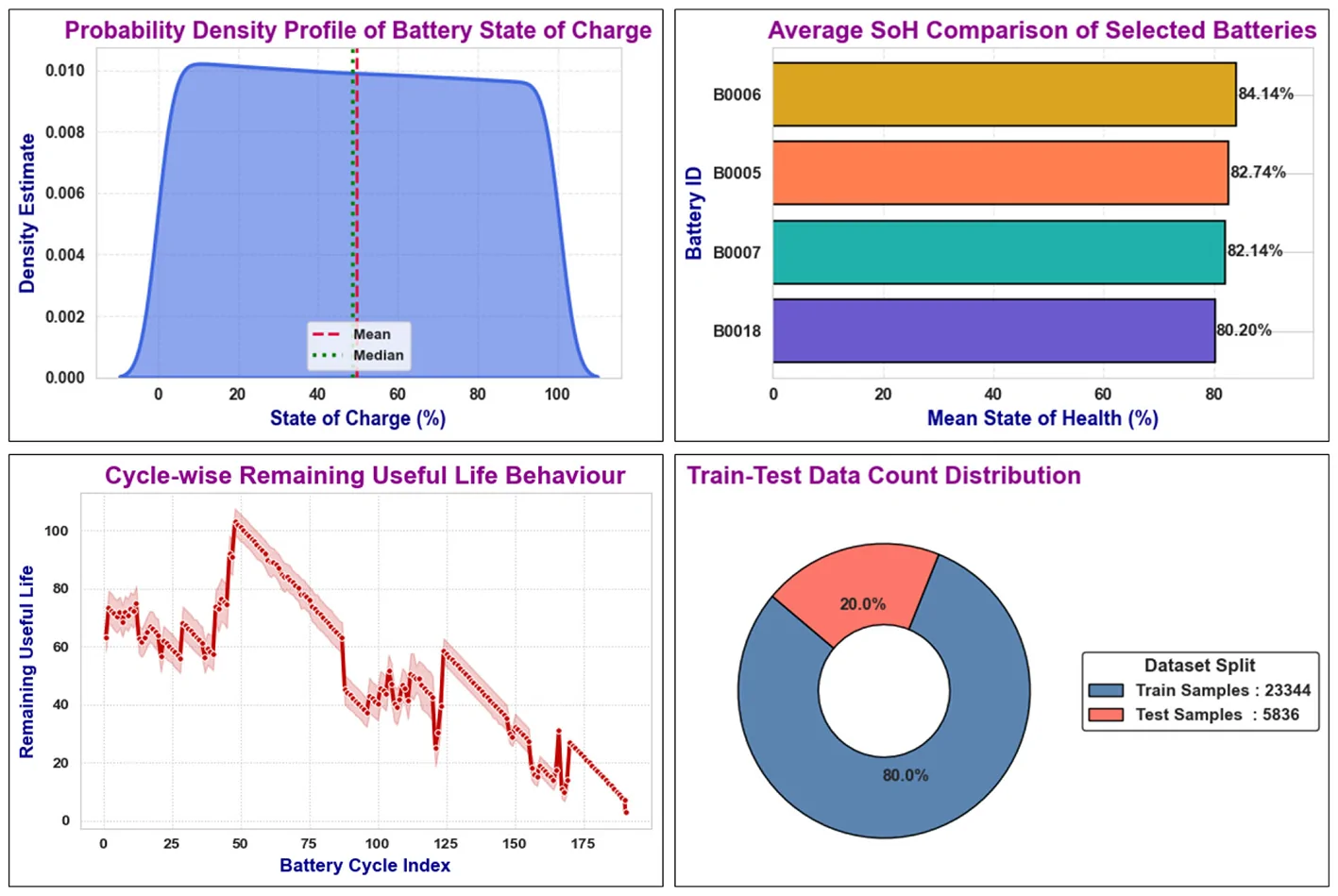
Battery performance analysis.

Figure 10 provides insight into the distribution of battery data and characteristics. When plotting the battery state-of-charge density, the distribution was uniform, with the mean and median at approximately 50%. SOH across varying states of charge was between approximately 80%−84%. The RUL curve shows a decrease with cycle index (battery cycles), indicating that all batteries eventually degrade over time; therefore, the RUL prediction decreases when calculating RUL for subsequent cycles. The dataset was split into a training and testing dataset and proportioned appropriately (80% for training and 20% for testing) to equalize the training and testing of the model.

In Table 9, the loss factors are determined manually, such as 0.35 for SOC, 0.45 for SOH, and 0.20 for RUL, depending on their significance and level of difficulty. Depending on the value, the proportion of effort devoted to it changes: the larger the value of a task, the more effort it receives, which means greater accuracy, although it may affect the performance of other tasks. The chosen weight values allow for satisfactory accuracy across all types of estimation.

**Table 9:** Loss weights of SOC, SOH, and RUL.

Parameter	SOC	SOH	RUL
Loss weight	0.35	0.45	0.20

**Figure 11:**
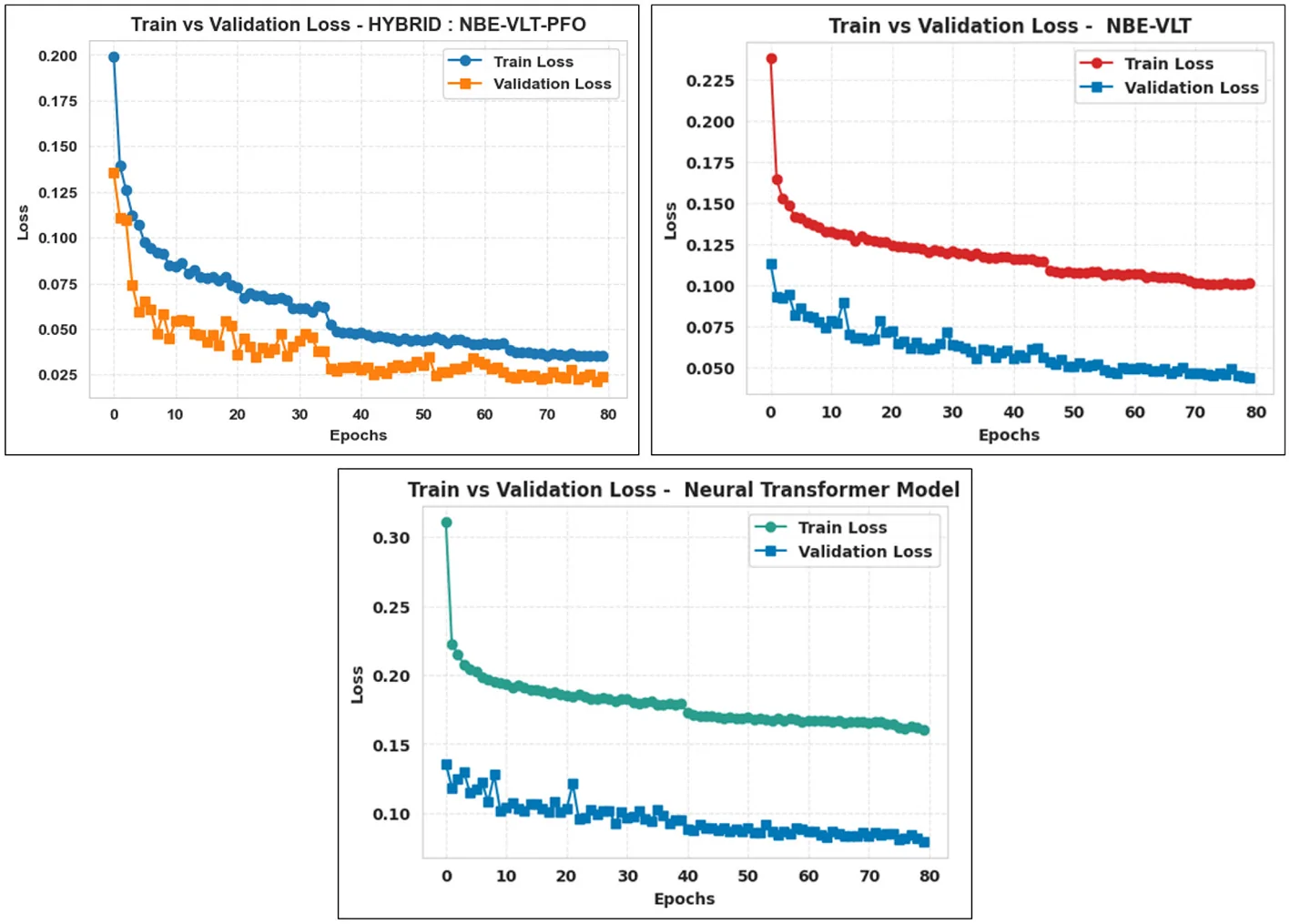
Train vs. validation loss of hybrid NBE-VLT-PFO, NBE-VLT, and neural transformer model.

The graph of loss curves in Figure 11 shows that all the models had reasonable learning curves over time. The loss values for training and validation for the Hybrid NBE-VLT-PFO model were consistently lower than the other models; thus, it outperformed the other two models in terms of performance and generalization. The NBE-VLT model had moderate loss values, whereas the Neural Transformer Model had the highest; therefore, it was the least effective of the three models.

**Figure 12:**
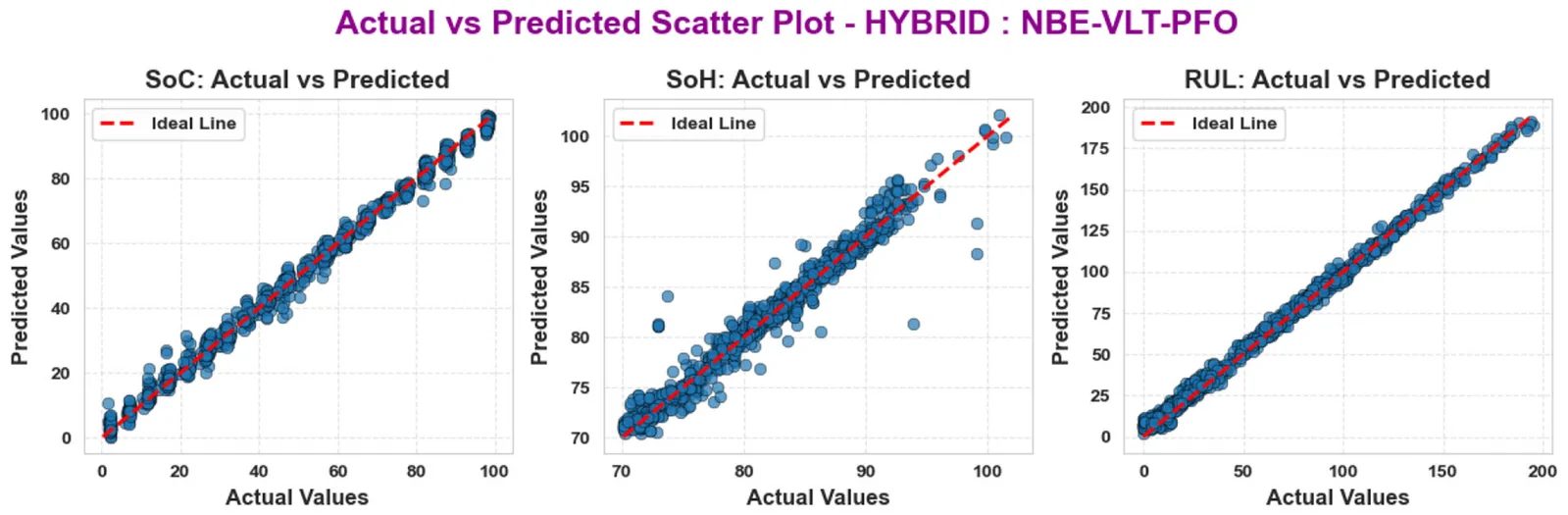
Actual vs. Predicted scatter plot of Hybrid NBE-VLT-PFO.

The scatterplots for the SOC, SOH, and RUL in Figure 12 show a very strong correlation among these metrics. The majority of points in each scatterplot cluster around the diagonal line representing the ideal regression line. This indicates that the Hybrid NBE-VLT-PFO model is the most accurate predictor of SOC, SOH, and RUL, with particularly strong performance in RUL and minimal error across all three battery metrics.

**Figure 13:**
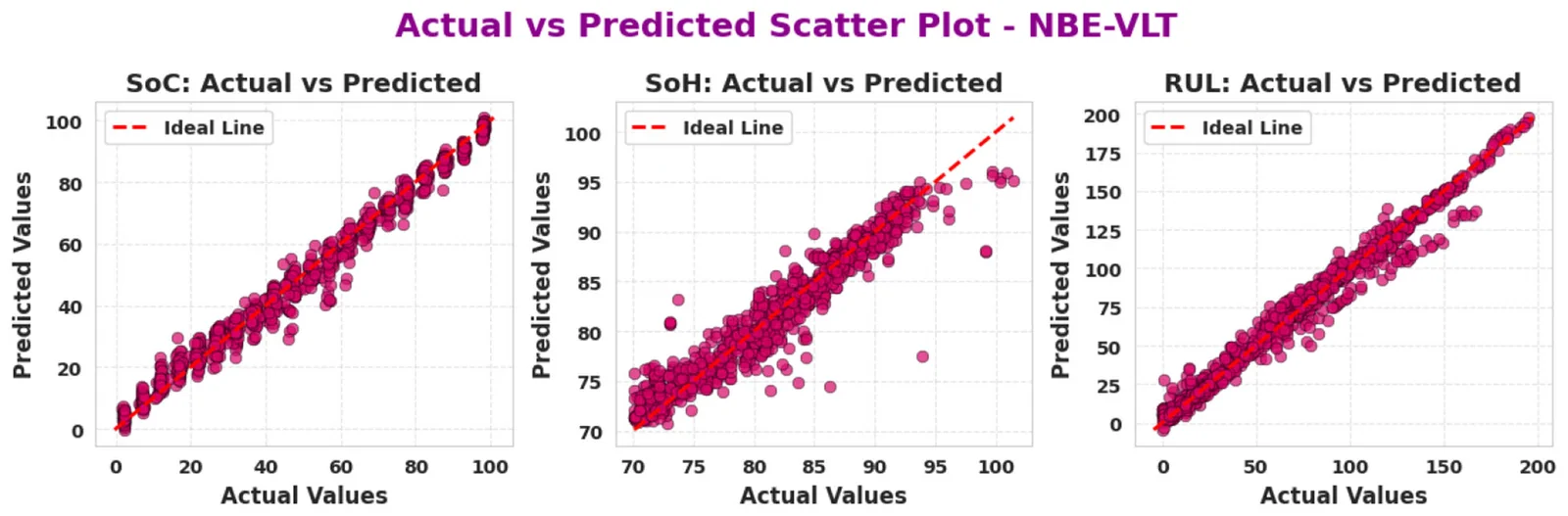
Actual vs. predicted scatter plot of NBE-VLT.

The scatterplots for the SOC, SOH, and RUL metrics in Figure 13 show that the NBE-VLT model replicated the actual test metrics for SOC, SOH, and RUL. However, the scatterplots for both SOH and RUL show greater variance in the predictive values of the NBE-VLT model than in those of the hybrid model, suggesting that the NBE-VLT model is more error-prone than the hybrid model.

**Figure 14:**
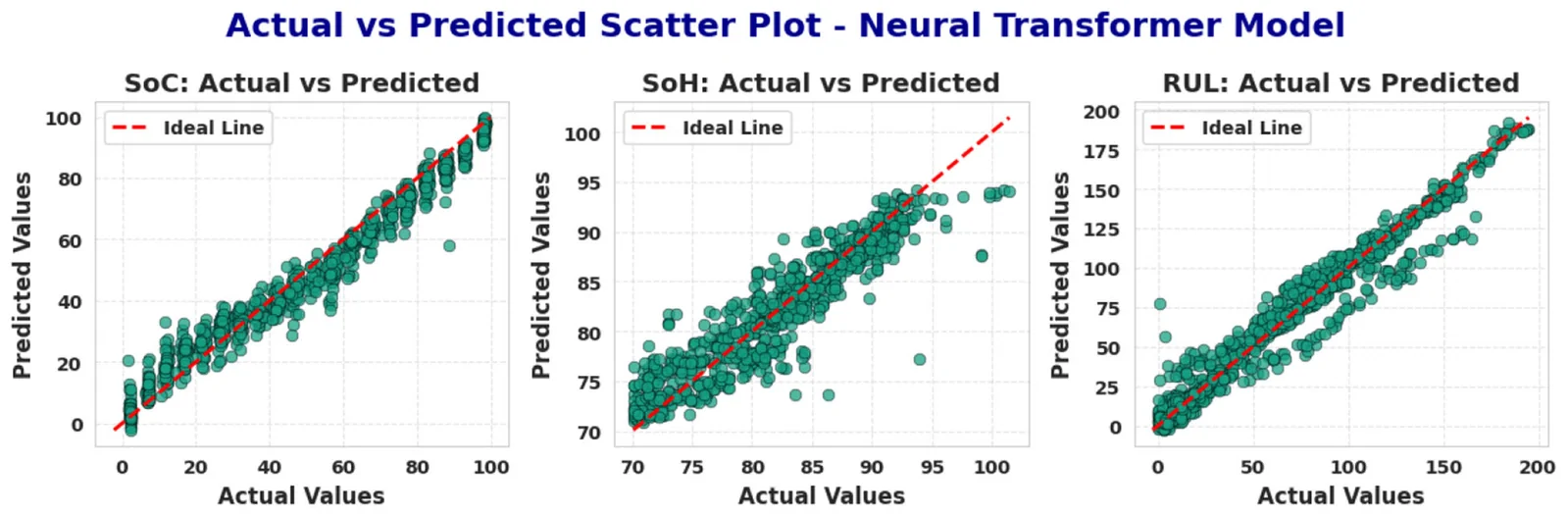
Actual vs. predicted scatter plot of neural transformer model.

The accuracy of the Neural Transformer model for prediction of SOC, SOH, and RUL is illustrated by the scatter plots in Figure 14. It shows that SOC and RUL have low scatter (near the ideal line) and high agreement between actual and predicted values, whereas the SOH prediction shows greater spread around the ideal prediction line and therefore has lower accuracy than either the SOC or RUL predictions.

In Table 10, the performance metric results obtained using the NASA battery dataset and CALCE battery dataset show the feasibility of the method for SOC, SOH, and RUL. The model achieved efficient accuracy results with the NASA dataset: the *R*^2^ score 0.9943 for SOC, 0.9606 for SOH, and 0.9936 for RUL estimation. The CALCE dataset performance results show *R*^2^ scores 0.9912, 0.9485, and 0.9891 for respective estimations.

**Table 10:** Performance evaluation on different public battery datasets.

Dataset	Target	MSE	RMSE	MAE	MASE	*R*^2^ score
NASA	SOC	0.0005	0.0225	0.0164	0.0128	0.9943
	SOH	0.0017	0.0414	0.0243	0.0189	0.9606
	RUL	0.0004	0.0196	0.0156	0.0117	0.9936
National Aeronautics and Space Administration (CALCE)	SOC	0.0008	0.0283	0.0205	0.0162	0.9912
	SOH	0.0024	0.0490	0.0301	0.0238	0.9485
	RUL	0.0007	0.0265	0.0198	0.0151	0.9891

The evaluation process in Table 11 uses different battery datasets such as NASA and CALCE. If the system is exposed to learning with the NASA dataset and tested on the CALCE dataset, it gives effective results, such as *R*^2^ are 0.9867, 0.9318, and 0.9825 for SOC, SOH, and RUL, respectively. When tested in reverse cases, the results of *R*^2^ values are 0.9908, 0.9547, and 0.9919.

**Table 11:** Cross-dataset generalization analysis.

Training dataset	Testing dataset	Target	MSE	RMSE	MAE	MASE	*R*^2^ score
NASA	CALCE	SOC	0.0010	0.0316	0.0234	0.0185	0.9867
		SOH	0.0031	0.0557	0.0342	0.0269	0.9318
		RUL	0.0011	0.0332	0.0247	0.0189	0.9825
CALCE	NASA	SOC	0.0007	0.0265	0.0191	0.0149	0.9908
		SOH	0.0020	0.0447	0.0275	0.0214	0.9547
		RUL	0.0006	0.0245	0.0182	0.0138	0.9919

**Figure 15:**
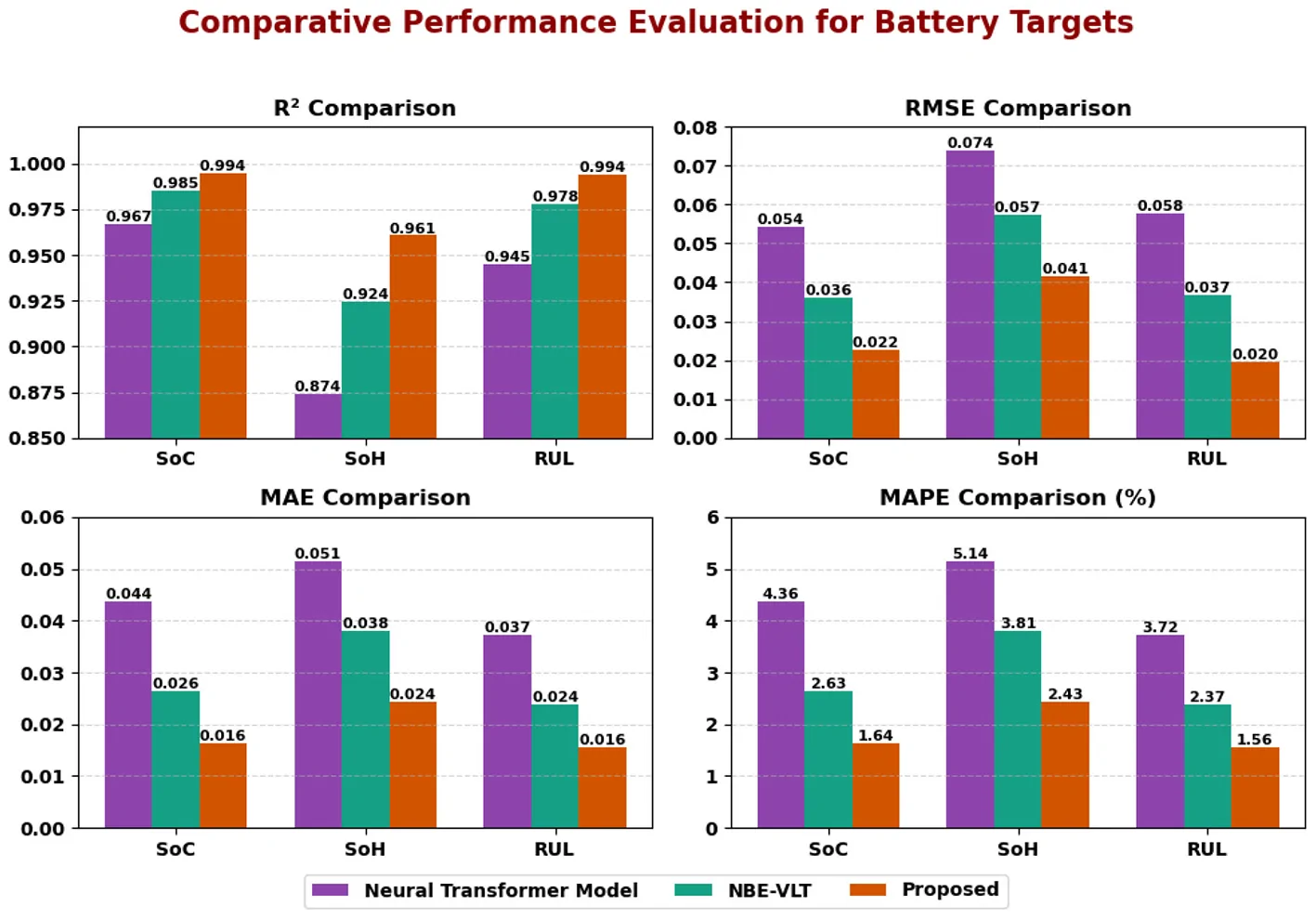
Comparison of evaluation metrics.

The comparative performance evaluation results in Figure 15 indicate that the suggested method steadily exceeded both the Neural Transformer model and the NBE-VLT models for all target variables such as SOC, SOH, and RUL. The proposed model achieved the greatest *R*^2^ values and the lowest RMSE, MAE, and MAPE, resulting in a much higher level of prediction accuracy, lower error, and higher reliability standards.

In Table 12, the proposed method overtook both the CNN-BiLSTM (Gao et al., 2023) and the RF-LSTM-XGBoost (Krishnasamy and Muniandi, 2026) methods with minimum MAE (0.0156) and RMSE (0.0196) values and maximum *R*^2^ (0.9936) value, demonstrating that the proposed method provides reliable estimates of lithium-ion battery RUL.

**Table 12:** Comparison of RUL predictions for estimating lithium-ion batteries.

S. No.	Methods	MAE	RMSE	*R* ^2^
1	CNN-BiLSTM (Gao et al., 2023)	0.5930	0.8306	0.9576
2	RF-LSTM-XGBoost (Krishnasamy and Muniandi, 2026)	0.025	0.035	0.96
3	Proposed	0.0156	0.0196	0.9936

**Figure 16:**
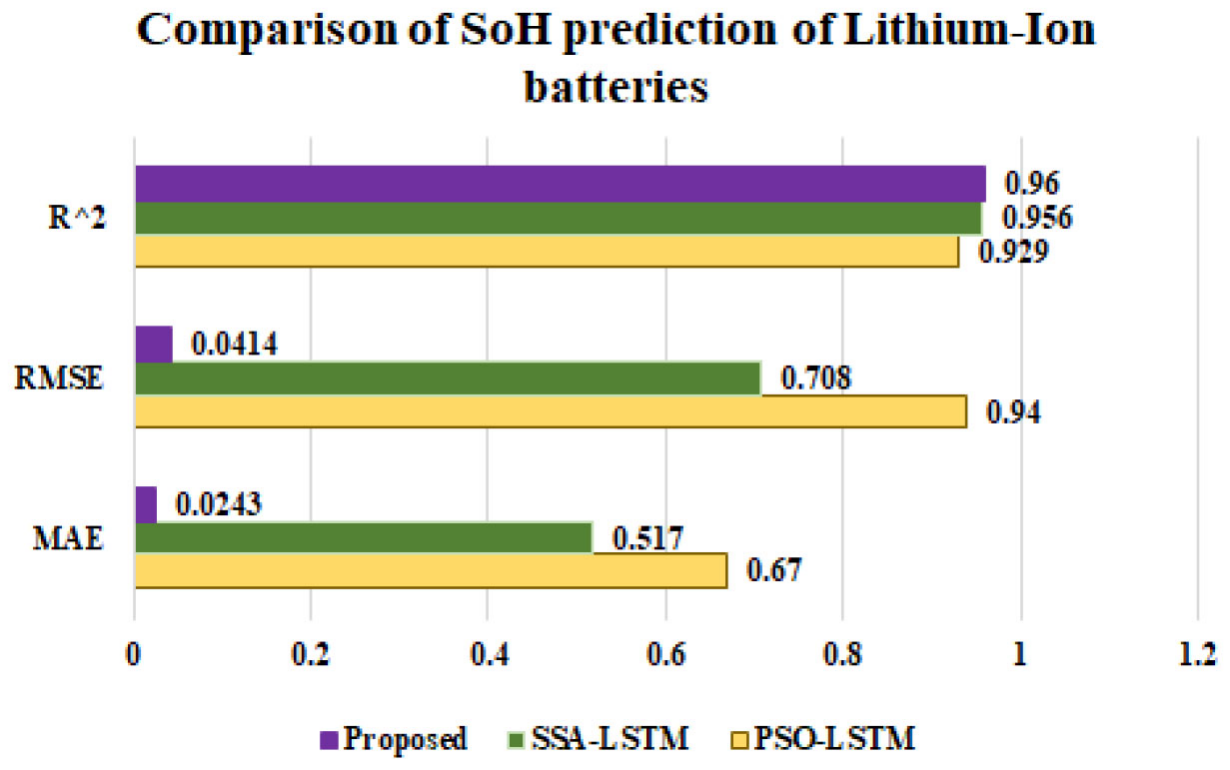
Comparative analysis of SOH prediction.

The SOH prediction on lithium-ion batteries in Figure 16 shows that the proposed technique ensures a stronger performance than SSA-LSTM (Li et al., 2025) and PSO-LSTM (Li and Chen, 2025). The maximum *R*^2^ (0.96), minimum RMSE (0.0414), and MAE (0.0243) values indicate that the suggested technique is significantly more precise than either of the other techniques.

SOC prediction of lithium-ion batteries in Table 13 indicates that the suggested process is greater compared to the Extra Trees model (Dini and Paolini, 2026) and GASVR (Chen et al., 2023), with lower MAE (0.0164) and RMSE (0.0225) and a higher *R*^2^ value (0.9943).

**Table 13:** Comparative analysis of SOC prediction.

S. No.	Methods	MAE	RMSE	*R* ^2^
1	Extra trees (Dini and Paolini, 2026)	0.0173	0.0367	0.982
2	GASVR (Chen et al., 2023)	0.77	0.95	0.9892
3	Proposed	0.0164	0.0225	0.9943

## 5 Conclusion

The NBE-VLT-PFO framework combines all data activities, including data preprocessing, hyperparameter optimization, hierarchical latent feature learning, Transformer-based temporal dependency modeling, and attention-based feature fusion. During the data preprocessing stage, data quality is improved through techniques such as feature encoding, outlier removal, feature analysis, and normalization. The PFO determines the optimal model configurations to improve convergence and the generalization ability of all models. Within the NBE, non-linear battery behavior is captured. At the same time, the Variational Ladder Encoder learns uncertainty-appropriate latent representations, and the Transformer Encoder learns to model the degradation pattern over time. The proposed model improves performance by incorporating fully connected regression layers, which yield accurate estimates of SOC, SOH, and RUL. The NASA battery dataset was used to evaluate the efficiency of the suggested architecture. For SOC, it provided *R*^2^ of 0.9943, RMSE of 0.0225, MAE of 0.0164, and MAPE of 1.64%. For SOH prediction, the model provided R^2^ of 0.9606, RMSE of 0.0414, MAE of 0.0243, and MAPE of 2.43%. Finally, for RUL prediction, the model achieved *R*^2^ of 0.9936, RMSE of 0.0196, MAE of 0.0156, and MAPE of 1.56%. These functional results show that the hybrid model is effective at providing accurate, reliable, and generalizable estimates of battery performance, thereby enabling predictive maintenance for EVs.

## Data Availability

Publicly available datasets were analyzed in this study. This data can be found here: https://drive.google.com/file/d/1H46hCYBJkGFz5MIT3ER4iy143CFkcyOh/view?usp=drivesdk.
